# Characterization of a *Ligilactobacillus salivarius* Strain Isolated from a Cheese Seal Which Was Last Used in 1936

**DOI:** 10.3390/foods13132005

**Published:** 2024-06-25

**Authors:** Claudio Alba, Rebeca Arroyo, Leónides Fernández, Arjan Narbad, Juan M. Rodríguez

**Affiliations:** 1 Department of Nutrition and Food Science, Complutense University of Madrid, 28040 Madrid, Spain; c.alba@ucm.es (C.A.); rebecaa@vet.ucm.es (R.A.); 2 Department of Galenic Pharmacy and Food Technology, Complutense University of Madrid, 28040 Madrid, Spain; leonides@ucm.es; 3 Food Microbiome and Health Institute Strategic Programme, Quadram Institute Bioscience, Rosalind Franklin Road, Colney, Norwich NR4 7UQ, UK; arjan.narbad@quadram.ac.uk

**Keywords:** cheese, cheese seal, *Ligilactobacillus salivarius*, adjunct culture, genome mining

## Abstract

Cheesemaking played a pivotal role in the life of the Pyrenean villages where cheese was a most prized commodity and the subject of much local competition. In one of them (Sasa de Sobrepuerto), Mrs. Sebastiana Palacio decided in 1877 to label all the cheeses made in her household with a seal to differentiate them from those made by other local producers. The cheese seal was last used in 1936 and, since then, it has been kept under excellent storage conditions. Since well-preserved cheese seals are rare, and bacterial cells may survive desiccation for long periods, the objective of this work was to isolate and characterize any lactic acid bacteria that survived in the seal. Analysis of the milky crust material revealed the presence of sheep caseins. Culture-based analysis led to the isolation of a strain of *Bacillus licheniformis* and a strain of *Ligilactobacillus salivarius* (*L. salivarius* SP36). The latter was characterized in vitro for safety and dairy-related functional properties. Its genome encodes several genes involved in protein, peptide, and amino acid catabolism, and flavor. Overall, the phenotypic and genetic features of this strain support a high potential for being used as adjunct culture in cheesemaking.

## 1. Introduction

Cheesemaking has been documented from, at least, the 6th millennium BC in Europe [1]. Such technology has been regarded as a milestone in human history due to the nutritional, cultural, and socio-economic impact of this activity [2]. The fact that cheese has a longer shelf life and is easier to transport than milk, together with its high nutritional value as a relevant source of proteins, fat, and calories, makes cheese as a very valuable food for surviving during cold seasons or periods of scarcity. It has been suggested that the introduction of this nutritious dairy product in the European diet decreased infant mortality rates among early farmers, enabling their demographic expansion [3].

Traditionally, cheesemaking was a key cultural and economic element in mountainous regions, such as the Spanish Pyrenees, where sheep constituted the main source of wealth until the 1950s–1960s of the last century. Cheese and cheesemaking played a pivotal role in the whole life cycle of the Pyrenean communities where “mountain cheese” was a most prized commodity and the subject of much local competition. Small changes in any of its properties, from its texture and shape to its flavor or cleanness, could lead to a particularly successful and highly prized cheese, highlighting the high skills of the farm where it was made. So, cheeses were a differential trait of each geographical zone, village, or even farm, a fact that is still evident under the different labels covering a wide variety of artisan cheeses in Europe: “Protected designation of origin” (PDO), Protected Geographical Indication (PGI), and Traditional Speciality Guaranteed (TSG) [4]. In other words, many cheeses are often heritage icons that remain deeply entwined with local and familiar culture and identity [5].

This was the case in a small region of the Spanish Pyrennees called “*Sobrepuerto*”, where cheeses were highly appreciated for centuries in Aragón. Sheep flocks moved (“*trashumaban*”) to lower lands between October/November and May/June. When they returned to the pastures around their villages in summer, the ewes’ milk was used to make their then, famous cheeses. All the villages within the Sobrepuerto region were completely uninhabited in the 1950s–1960s of the 20th century. In one of them (Sasa de Sobrepuerto), one woman (Mrs. Sebastiana Palacio, born in 1853 in Sarsa de Surta, Huesca) decided in 1877 to label all the cheeses made in her house (Casa Juan Domingo) with a seal containing her initials since she was proud of her skills and hygienic procedures during cheesemaking. Such a practice lasted until 1936, when the last owners of the house (Mr. José Villlacampa and Mrs. Julia López) decided to move to a bigger village (Fiscal, Huesca), and to give up sheep farming after their ovine flock was requisitioned during the Spanish Civil War.

A few years ago, we discovered the existence of the cheese seal, which had been kept by a direct descendent (Mrs. Anabel Puyuelo, Fiscal), under excellent storage conditions (original box, cold and dark place). The seal was covered by a homogeneous dairy crust. Since such well-preserved cheese seals are rare, and vegetative bacterial cells may survive desiccation for long periods, particularly when protected by milky environments [6,7,8,9], the objectives of this work were, first, to submit the dairy crust to an enrichment step in order to know if culturable lactic acid bacteria remained in the seal and, if it was the case, then undertake a preliminary in vitro and in silico characterization of the isolated strain(s). In parallel, a metataxonomic analysis of this old biological material was also performed to explore which bacterial species might have been originally present in the seal.

## 2. Materials and Methods

### 2.1. In Search for the Seal

During an interview with Antonio Bellosta (born in 1914 in Naval, Huesca, Spain), who worked as a muleteer in the Spanish Pyrenees from the late 1920s to the early 1950s of the last century, he reported that he used to acquire cheeses, which were usually exchanged with olive oil (approximately 100 kg of cheeses with 20 L of olive oil), during his regular trips to the region called “*Sobrepuerto*”. The cheeses were derived from milk collected from local ewes (Churra tensina breed) using rennet that had been obtained directly from the stomach of a lactating lamb. The cheeses were prepared via a back-slopping fermentation and matured for at least 6 months. Among the different local cheese producers, he remembered that the ones with the best organoleptic properties, and therefore, reaching the highest demand and economic value among his clients, were those made in Casa Juan Domingo (Sasa de Sobrepuerto, Huesca). In addition, they were uniquely labeled with a seal or stamp (“*a couple of letters*”) on their upper surface. Some months later, and in an independent interview, Mrs. Angelines Villacampa (Casa Mallau, Susín, Huesca) informed that her mother (Mrs. Avelina Villacampa) was born in Casa Juan Domingo. Mrs. Villacampa said that, during her infancy, she used to spend summers in her mother`s village where she witnessed cheesemaking by her grandmother, her mother, and other women living in the same house. She confirmed the existence of a cheese seal used to label all the cheese pieces produced in the household. In addition, she said that Mrs. Anabel Puyuelo, a daughter of her cousin Joaquín Puyuelo, kept the seal in her house in Fiscal (Huesca), the village where the family settled when they left Sasa de Sobrepuerto in 1936.

**Figure 1:**
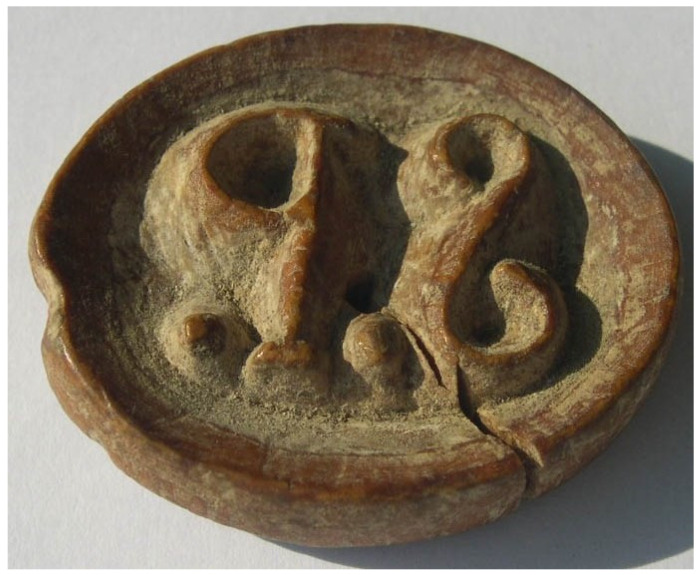
The seal created in 1877 to label the cheeses made by Mrs. Sebastiana Palacio in Sasa de Sobrepuerto (Huesca, Spain). The seal was uninterruptedly used to label all the cheeses produced in Casa Juan Domingo since then and until 1936.

In the subsequent visit to Mrs. Puyuelo, she showed us the seal. It was a wooden oval, measuring 4.3 × 3.6 × 0.6 cm, which had been carved to carry the negative of two letters (“S.P.”, after Sebastiana Palacio) (Figure 1). The external surface of the seal conserved a dairy crust. It has been conserved inside a crystal box in a dry and dark cupboard since it was last used in the summer of 1936.

### 2.2. Collection of Biological Material from the Seal

The whitish milky crust present in the external surface of the seal was carefully removed using a scalpel and a sterile swab soaked in sterile peptone water. Then, the material was diluted in 3 mL of sterile peptone water.

### 2.3. ELISA Detection of Ovine- and Bovine-Specific Caseins and β-Lactoglobulins

The biological material obtained from the external surface of the seal was analyzed for the presence of either ovine- or bovine-specific caseins and β-lactoglobulins using a quantitative sandwich ELISA approach. In the case of caseins, the sheep casein alpha (CSN1) and the bovine casein alpha (CSN1) ELISA kits were employed (MyBioSource, Inc., San Diego, CA, USA). The sensitivity of the sheep casein-targeting kit was 5.0 pg/mL (detection range: 31.2–1000 pg/mL) while that of the cow casein-targeting kit was <7.5 pg/mL (detection range: 15.6–1000 pg/mL). In parallel, the sample was also assayed for the presence of either ovine- or bovine-specific β-lactoglobulins using the sheep beta-lactoglobulin and the bovine beta-lactoglobulin ELISA kits, respectively (MyBioSource, Inc.). In these cases, the sensitivity of the sheep-targeting kit was 0.1 mg/mL (detection range: 0.25–8 mg/mL) while that of the cow-targeting kit was 0.067 ng/mL (detection range: 156–10 ng/mL). To perform these analyses, an aliquot of the sample (0.5 mL) was centrifuged for 15 min at 1500× *g* and the supernatant was collected and immediately assayed.

### 2.4. Culture of the Biological Material

Proper dilutions of an aliquot (0.5 mL) of the seal-derived material were spread onto plates of the following media: MacConkey (MCK, BioMérieux, Marcy l’Etoile, France, isolation of enterobacteria), Columbia Nadilixic Acid (CNA, BioMérieux, streptococci, enterococci, staphylococci, and related Gram-positive bacteria), Sabouraud Dextrose Chloramphenicol (SDC, BioMérieux; yeasts), Polymyxin Pyruvate Egg yolk Mannitol Bromothymolblue Agar (PEMBA, BioMérieux; *Bacillus*), and De Man, Rogosa and Sharpe (MRS, Oxoid) supplemented with 0.05% (*w*/*v*) l-cysteine and bromophenol blue (MRS-Cys; isolation of lactic acid bacteria and bifidobacteria). CNA, MCK, SDC, and PEMBA plates were incubated aerobically at 32 °C for 48 h while MRS-Cys plates were incubated in anaerobiosis (85% nitrogen, 10% hydrogen, 5% carbon dioxide) at 37 °C for 48 h. A second aliquot (0.5 mL) was used for enrichment in Brain Heart Infusion (BHI) broth (25 mL) for 48 h at 32 °C. Then, serial dilutions of the enriched broth were spread on the same agar media described above and the plates were incubated under the same conditions. The isolates, including at least one representative of each colony morphology, were identified by both 16S rDNA sequencing [10] and MALDI-TOF mass spectrometry [11]. The only isolate that belonged to the lactic acid bacteria group was *Ligilactobacillus salivarius* SP36, which was submitted to the characterization of some properties related to the safety and functionality of cheesemaking.

### 2.5. In Vitro Characterization of L. salivarius SP36

The scheme used for the assessment of some features of *L. salivarius* SP36 was similar to that previously used by our group for the characterization of *L. salivarius* CECT 9145 and *L. salivarius* PS7, two strains belonging to the same species [12,13]. All the assays were carried out in triplicate. The antimicrobial activity of the strain against pathobionts and spoilage microbes that are prevalent in cheese environments was assayed, employing an overlay method [14,15]. The indicator strains included the bacteria *Clostridium tyrobutyricum* CECT 4011, *Staphylococcus aureus* CECT 976, *Staphylococcus aureus* CECT 5191, *Staphylococcus epidermidis* CECT 231, *Listeria monocytogenes CECT* 936, *Listeria monocytogenes CECT* 4031, *Salmonella enterica* subsp. *enterica* serotype Enteritidis *CECT* 4300, *Salmonella enterica* subsp. *enterica* serovar Typhi CECT 409, *Salmonella enterica* subsp. *enterica* serovar *Typhimurium CECT* 443, *Escherichia coli* CECT 4076, *Escherichia coli* CECT 4076 (O157:H7), *Klebsiella pneumoniae* CECT 142, *Klebsiella oxytoca* CECT 860 and *Proteus vulgaris* CECT 484, the molds *Aspergillus fumigatus* CECT 2071 and *Penicillium commune* CECT 20707, and the yeasts *Rhodotorula mucilaginosa* CECT 10359, *Kluyveromyces marxianus* CECT 10357, *Candida intermedia* CECT1431, and *Debaryomyces hansenii* CECT10360. All the indicator strains were provided by the Spanish Collection of Type Cultures (CECT, Burjassot, Spain). The plates overlaid with bacterial indicators were incubated at 32 or 37 °C (depending on the optimal temperature) for 48 h, while those overlaid with yeasts cells or fungal spores were incubated at 30 °C (yeasts, molds) for 48 h (yeasts) or 120 h (molds). Finally, the plates were examined for clear zones of inhibition (>2 mm) around the *L. salivarius* SP36 streaks.

Potential bacteriocinogenic activity against the bacterial indicator strains was assessed as described previously [15]. l- and d-lactic acid concentrations in the MRS supernatants of *L. salivarius* SP36 (obtained after an incubation at 37 °C for 16 h) were assayed enzymatically using the Roche Diagnostics (Mannheim, Germany) kit. Their pH values were also determined. The concentration of both lactic acid enantiomers and the pH were also quantified in UHT milk (pH 6.5) inoculated with the strain (approximately 10^6^ cfu/mL) following the same procedure. Hydrogen peroxide production was qualitatively and quantitatively assayed using previously described methods [16,17], including the modifications proposed by Martín et al. [15].

Potential co-aggregation between *L. salivarius* SP36 and the bacterial and yeast indicator strains, its bacteriocinogenic activity against the bacterial indicator strains, and its capacity to adhere to intestinal (Caco-2 and HT-29) epithelial cells were assessed as described previously [15]. Adhesion of the probiotic strain to porcine mucin was assayed as described [18].

The capability of *L. salivarius* SP36 to survive after exposition to oral and gastrointestinal-like conditions was evaluated. For this purpose, the methodology proposed by Marteau et al. [19], and modified by Martín et al. [15], was selected.

The susceptibility of *L. salivarius* SP36 to antibiotics was tested using the E-test system (Biomerieux, Marcy l’Etoile, France) and the antibiotics (ampicillin, clindamycin, chloramphenicol, erythromycin, streptomycin, gentamicin, kanamycin, and tetracycline) and cut-off values proposed for this species by EFSA [20]. In addition, the strain was also examined for hemolysis when grown on horse blood agar medium [12], for biogenic amines biosynthesis (putrescine, cadaverine, tyramine, and histamine) [21], and for its potential for gastric mucin (HGM; Sigma, Kawasaki City, Japan) degradation [22].

### 2.6. Metataxonomic Analysis of the Milky Crust of the Seal

For the study of the seal microbiota, 1 mL of the material extracted from the milky crust was used for DNA extraction. This process followed a previously established method [23]. The extracted DNA was eluted in 22 µL of nuclease-free water and immediately stored at −20 °C until further analysis. The purity and concentration of the DNA were estimated using a NanoDrop 1000 spectrophotometer (NanoDrop Technologies, Inc., Rockland, DE, USA). To prevent contamination, a negative control was processed in parallel with the sample.

The V3-V4 hypervariable regions of the 16S rDNA were amplified by PCR using universal primers S-D-Bact-0341-b-S-17 (CCTACGGGNGGCWGCAG) and S-D-Bact-0785-a-A-21 (GACTACHVGGGTATCTAATCC) [24]. The PCR products were sequenced on the Illumina MiSeq system at the facilities of Parque Científico de Madrid (Tres Cantos, Spain). Barcodes were added to both the 3′ and 5′ terminal ends of the PCR amplicons to facilitate the separation of forward and reverse sequences in a subsequent PCR reaction. The DNA concentration of the PCR products was quantified using a 2100 Bioanalyzer system (Agilent, Santa Clara, CA, USA). After pooling the PCR products at equal molar ratios, the DNA amplicons were purified using a QIAEX II Gel Extraction Kit (Qiagen, Hilden, Germany). The DNA concentration was then quantified with PicoGreen (BMG Labtech, Jena, Germany). The pooled, purified, and barcoded DNA amplicons were finally sequenced using the Illumina MiSeq pair-end protocol following the manufacturer’s guidelines.

Raw sequence data from the cheese seal sample were demultiplexed and filtered using Illumina MiSeq Reporter v2.6 analysis software. Microbiome bioinformatics was performed using QIIME 2 (2022.2), performing sequence denoising via DADA2 to enhance data accuracy. Taxonomic classifications were assigned using the q2-feature-classifier and the naïve Bayes classifier classify-sklearn, based on the SILVA database version 138.1. Further bioinformatic analysis was conducted in R (version 4.3.2), generating a table of Amplicon Sequence Variants (ASVs) counts. Bacterial taxa abundances were normalized utilizing the total sum scaling (TSS) method.

### 2.7. Whole Genome Sequencing and Mining of L. salivarius SP36

A bacterial culture of the *L. salivarius* SP36 was grown to a stationary phase over a period of 1–3 days and then harvested by centrifugation (1.5 mL at 13,000 rpm for 2 min). The pellet was resuspended in 1 mL of TE buffer (10 mM Tris-HCl, pH 8.0, 1 mM EDTA), centrifuged again, and the resultant pellet was frozen. For enzymatic lysis, the pellet was resuspended in 300 µL of TE buffer supplemented with 100 µL of lysozyme (25 mg/mL), 1–3 µL of mutanolysin (10 U/µL), and 1 µL of RNase A (10 mg/mL), and incubated at 37 °C for 30 min. DNA was extracted using the Maxwell^®^ RSC Cultured Cells DNA Kit (Promega, Madison, WI, USA) according to the manufacturer’s instructions. Libraries were created using the Nextera XT DNA Library Preparation Kit (Illumina, San Diego, CA, USA) and sequenced on a NextSeq 550 System (Illumina) producing 150 base pair (bp) paired end reads, as previously described [25]. Whole genome sequences of *L. salivarius* SP36 were deposited in GenBank under the accession number SAMN40261862.

The genomic sequence reads were processed using an integrated bioinformatics pipeline. Initial quality control was carried out using Fastp (Version 0.19.5) [26], which refined the read quality using adapter trimming and quality-based filtering. Taxonomic classification was performed with Kraken2 (Version 2.1.1 [27], utilizing the ‘k2_nt_20230502’ database to ensure precise taxonomic resolution at minimal confidence thresholds.

Subsequent de novo assembly of the quality-filtered reads was conducted using Shovill (Vr. 1.1.0) [28]. Genomic annotation was performed with RAST and Bakta, optimized for bacterial genomes to enhance annotation accuracy. Assembly integrity was evaluated with CheckM (Vr. 1.0.11) [29], while plasmid content was predicted with PlasFlow (Vr. 1.0) [30]. The assembled contigs were submitted to virulence factor analysis against the ‘vfdb_full’ database, antimicrobial resistance gene detection with ABRicate (Vr. 0.9.7) [31], and qualitative assessment through Quast (Vr. 5.0.2) [32]. Prophage regions were detected using Phigaro, a high-throughput prophage sequence application [33] while AntiSMASH (v. 7.0) was used for the mining of bacteriocin-related genes.

## 3. Results

### 3.1. ELISA Detection of Ovine- and Bovine-Specific Caseins and β-Lactoglobulins

In this study, the ELISA technique was used for the detection of ovine- and bovine-specific caseins and β-lactoglobulins in the milky crust present on the surface of the seal. Cow proteins and sheep β-lactoglobulin were below the detection limit of the assay. In contrast, the concentration of ovine casein was 229 pg/mL, a value that was within the detection range of the technique.

### 3.2. Isolation and In Vitro Characterization of L. salivarius SP36

**Figure 2:**
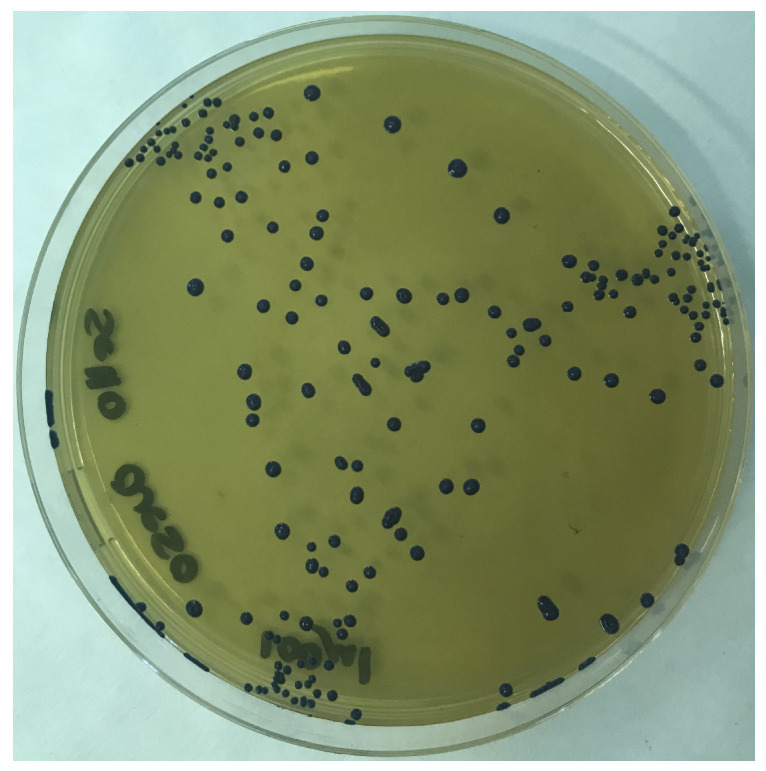
Colonies obtained on MRS-Cys agar plates after the BHI enrichment step.

Bacterial growth from the dairy crust was only achieved after the enrichment step in BHI broth. When this broth medium was cultured on agar plates, growth was only obtained in PEMBA and MRS-Cys plates. Only one type of colony was observed in each medium. The ones that grew on PEMBA plates were identified as *Bacillus licheniformis* while those grown on MRS-Cys plates were identified as *L. salivarius* (Figure 2).

*L. salivarius* SP36 showed antimicrobial activity against all the indicator microorganisms used in this work, except for *D. hansenii* CECT 10360 (no inhibition zone). The inhibition zone was large (>3 mm around the streak) against the *C. tyrobutyricum* strain and the Gram-negative bacterial indicators, medium (2–3 mm) against the remaining Gram-positive bacteria, and narrow (<2 mm) against the molds and the remaining yeasts. To elucidate the compound(s) responsible for the antimicrobial activity, the strain was screened for the production of bacteriocins, hydrogen peroxide, and lactate. Bacteriocin activity could not be detected against the spectrum of indicator organisms used in this study while it showed an ability to produce H_2_O_2_ (0.78 μg/mL ± 0.12). The l-lactic acid production by *L. salivarius* SP36 when grown in MRS broth for 16 h at 37 °C was 11.09 ± 0.87 mg/mL, which corresponded with a mean pH value of 3.94. In contrast, the amount of L-lactate detected in the milk cultures was much lower (0.21 ± 0.04 mg/mL, pH = 5.97). d-lactic acid was detected neither in the MRS supernatants nor in the milk cultures of the strain.

The strain displayed a co-aggregation ability against all the bacterial and yeast indicators, which was particularly intense against the *L. monocytogenes* strains and against the yeasts *R. mucilaginosa* CECT 10359 and *C. intermedia* CECT 1431. Its adherence to HT-29 and Caco-2 cells was high since it showed means of 371.2 ± 46.0 and 333 ± 39 adhered lactobacilli cells in 20 random microscopic fields, respectively. Adhesion of the strain to porcine mucin (12.3% ± 1.4 of retained fluorescence) was also high. The viability of the strain after exposition to oral and gastrointestinal-like conditions was ~63.7%.

The MIC values of *L. salivarius* SP36 for the antibiotics tested in this study using the E-test procedure revealed that the strain was sensitive to all of them, with MIC values that were acceptable according to the breakpoints proposed by EFSA [20]. The only exception was kanamycin, but this is an intrinsic feature of the *L. salivarius* species. Concerning other safety-related properties, the strain lacked hemolytic activity, and neither degraded gastric mucin nor produced biogenic amines.

### 3.3. Metataxonomic Analysis of the Milky Crust of the Seal

**Figure 3:**
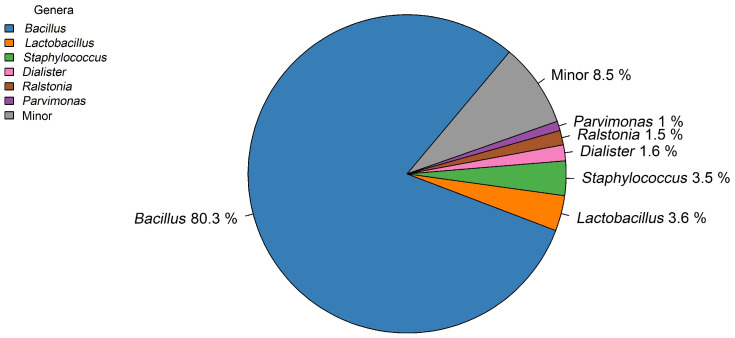
Pie chart with the relative abundance of microbial genera in the milky crust of the seal.

In the metataxonomic analysis of the sample collected from the seal, a total of 66,116 high-quality sequences were obtained. The analysis revealed a low diversity composition since most of the sequences (91.5%) belonged to only six genera. The most abundant genus was *Bacillus* (80.3% of the retrieved sequences) followed by, although at much lower percentages, *Lactobacillus* (3.6%), *Staphylococcus* (3.5%), *Dialister* (1.6%), *Ralstonia* (1.5%), and *Parvimonas* (1%) (Figure 3). The rest of the genera detected in the sample (minor genera) collectively accounted for 8.5% of the total sequences.

### 3.4. Genome Sequencing and Mining of L. salivarius SP36

The genome of *L. salivarius* SP36 comprises 1,964,896 base pairs, with a GC content of 32.83%, and includes 1968 coding sequences. These sequences are further categorized into 210 subsystems as analyzed by the RAST server. None of the genes found in its genome had a similarity with any known gene involved in virulence or pathogenicity. Some genes that may be involved in functionality, safety, stress resistance, and cheesemaking performance were detected in the genome of *L. salivarius* SP36 (Table 1).

The strain harbors l-lactate dehydrogenase genes (*ldhL*) and may metabolize lactose predominantly through the Leloir pathway with alpha-galactosidase, a N-acetylgalactosamine permease IID component, galactokinase, galactose-1-phosphate uridylyltransferase, and aldose 1-epimerase as the featuring key enzymes. Additionally, a galactose operon repressor from the GalR-LacI family of transcriptional regulators, playing a crucial role in metabolic regulation, was also detected. The strain did not contain genes encoding caseinolytic proteinases, like serine proteases but, in contrast, the genome encodes a complex array of genes involved in the catabolism of other proteins and peptides, including various proteases, endo-, amino-, and carboxypeptidases, as well as enzymes responsible for amino acid catabolism and flavor (Table 1). Notable among these are the aromatic amino acid aminotransferase gamma and quinate 5-dehydrogenase I beta. Additionally, the genome features several genes related to lipolysis, including cyclic-di-AMP phosphodiesterase, phosphoesterase, thioesterase, and glycerophosphoryl diester phosphodiesterase.

Additionally, the search tool Antismash identified two regions potentially involved in bacteriocin production. The first region is classified as a class I lanthipeptide, showing a 63% similarity to the known nisin cluster, a member of the RiPPs (ribosomally synthesized and post-translationally modified peptides) within the lanthipeptide group of bacteriocins. However, the similarity rate with the nisin gene cluster was relatively low (63%, 63%, and 66% with respect to nisin Q, nisin A, and nisin Z, respectively), and two key genes for nisin biosynthesis (the one encoding the leader peptidase and the immunity gene) were lacking in the genome of our strain. The second region is characterized as a RiPP-like peptide, exhibiting a 66% similarity to the salivaricin CRL1328 α and β peptides. The strain also contains genetic determinants enabling the production of hydrogen peroxide, another compound with antimicrobial activity.

The presence of the *bsh* gene may contribute to its resistance when exposed to human gastrointestinal-like conditions while the presence of several genes involved in different types of stress may help to explain its survival for decades in the cheese seal.

The genome includes phage-related open reading frames scattered across the chromosome, alongside an entire prophage sequence detected by the Phigaro tool, encompassing a DNA segment of 23.8 kb identified as a *Siphoviridae* prophage.

## 4. Discussion

In this work, we isolated an *L. salivarius* strain from the biological remains that existed on the surface of an old cheese seal. This seal was used uninterruptedly during all the summers from 1877 to 1936. It was made of wood (with small pores and fine cracks) and remained at least 8 months a year covered by a dried milk layer. These factors favored the long-term survival of some of the microbes that inhabited and were able to remain alive within the desiccation-adapted biofilms [9]. It has been shown that some microbes, including foodborne pathogens, can resist drying processes and survive for long periods in low-moisture foods and environments, especially after exposure to non-lethal stress conditions, such as sub-lethal acidic or salty environments, which are present during cheese production, and, eventually, can grow if the dry food is rehydrated [6,7]. As an example, some species of the genus *Acinetobacter*, which are not particularly resistant to environmental conditions, can maintain their viability in desiccated powdered infant formula for two years, which is often the whole shelf-life of these products; *Acinetobacter* spp. are able to maintain their viability in desiccated infant formula for 2 years [8].

The culture-based assessment of the seal-derived sample led to the growth of isolates belonging to two bacterial species (*B. licheniformis* and *L. salivarius*). Interestingly, the metataxonomic analysis revealed that the genus *Bacillus* (80.3%) and the genus *Lactobacillus* (3.6%) were the two most abundant genera in the seal sample. It must be considered that *Lactobacillus* sequences in this metataxonomic analysis include all the genera in which this genus was reclassified in 2020 [44], including the genus *Ligilactobacillus*. The genus *Bacillus* includes spore-forming species and some of them (*B. licheniformis*, *B. cereus*, etc.) are often found in dairy farm environments (soil, feed, udders, and milk) [45,46]. They can also be frequently found in artisanal and commercial ewe’s milk cheese [47,48]. The high resistance of *Bacillus* spp. to environmental conditions may explain why this was the most abundant genus in the metataxonomic analysis. In 2006, one strain of *B. licheniformis* and others of *Bacillus subtilis* were isolated from milk powder taken to Antarctica on Shackleton’s expedition in 1907 [49].

In relation to the presence of *L. salivarius* in the seal sample, this species has been previously isolated from ovine biological samples, including sheep dung [50] and the rumen of a grazing lamb [51], and also from milk of different mammalian species [15,52,53,54,55,56]. Therefore, it seems to be a species that is particularly well adapted to milk and mammary environments. The resistance of *L. salivarius* strains to spray-drying depends largely on the strain and the process parameters [57] but some strains show high survival rates after drying (>94%) and they remain stable (>80% survival) for several months [58]. Anyway, this work is, to our knowledge, the first in which an isolate of this species, or any other lactic acid bacteria species, has been recovered after several decades (more than 80 years) from its last use. The fact that they have been conserved at a relatively constant temperature (approximately 12–16 °C) and in the dark throughout this period, together with the presence of several genes related to different types of environmental stress in the genome of *L. salivarius* SP36, may have helped them to maintain the viability of a small percentage of the original bacterial load.

The metataxonomic analysis also detected DNA sequences at an abundance of ≥1% from the genera *Staphylococcus*, *Ralstonia*, *Dialister*, and *Parvimonas*. The genus *Staphylococcus* is the most abundant one in the core microbiota of milk from either healthy or mastitic ewes [59,60]. So far, there are no reports describing the presence of *Ralstonia*, *Dialister*, or *Parvimonas* DNA in ovine milk samples, although, *Ralstonia* sequences have been found in donkey and cow milk [61,62].

WGS of strains isolated from artisanal cheeses provides useful information for the selection of potential starter or adjunct cultures to recreate, at least to some extent, the organoleptic properties of the traditional cheeses [63]. In addition to safety-related issues, the production of antimicrobial substances and the metabolic and enzymatic activities responsible for acidification or sensorial properties are traits of particular interest to the dairy industry [64,65]. In this work, *L. salivarius* SP36 displayed noticeable inhibitory activity against dairy-related microbes that may cause spoilage or foodborne disease. Such activity seems to be due to the in vitro production of high amounts of l-lactate and small amounts of hydrogen peroxide. This strain harbors genes that are associated with the production of such compounds. In contrast, this activity was not due to the biosynthesis of bacteriocins. The strain contains gene information related to the bacteriocins nisin and salivaricin; however, it lacks some genetic elements that are essential for their expression or transport, and, therefore, the strain is unable to produce these antimicrobial peptides. In previous work, the analysis of the genome of *L. salivarius* CECT 5713 showed that it contained all the genes required for the biosynthesis of salivaricin Abp118, with almost a 100% identity with respect to the same genes present in the genome of the salivaricin-producing strain *L. salivarius* UCC118. The only significant difference was related to the *abpK* gene, which encodes the histidine kinase determinant involved in the *quorum sensing* system required for the regulation of bacteriocin synthesis in strain UCC118. More specifically, there is a four nucleotide deletion at the beginning of the *abpK* gene of CECT 5713, which introduces a frameshift mutation resulting in a premature stop codon, resulting in the absence of bacteriocinogenicity [39].

The strain lacks genes encoding caseinolytic proteinases, a fact that may explain its low growth and poor acidification when inoculated in milk, which may prevent its use as a starter culture. In contrast, the genome encodes a complex array of genes involved in protein and peptide degradation, amino acid catabolism, and flavor. Overall, these phenotypic and genetic features support the high potential of this strain for being used as adjunct culture in cheesemaking.

## 5. Conclusions

The results of this work show that old cheese-related biological materials can contain viable bacteria that can be recovered after an appropriate enrichment step. Such isolated strains were routinely employed through back-slopping before the generalization of the use of commercial starter cultures. They may display interesting functional and technological properties enabling their use as starter or adjunct cultures for local producers willing to recover traditional organoleptic properties. In this study, *L. salivarius* SP36 proved to be safe and to have some functional properties of interest for dairy companies. The analysis of its genome indicated that it is not a good candidate as a starter culture but an excellent one as an adjunct culture for cheesemaking.

## Figures and Tables

**Table 1:** Genes detected in the genome of *L. salivarius* SP36 that may be involved in functionality, safety, stress resistance, and cheesemaking performance.

**Category**	**Peptide or Protein-Encoding Genes**	**Brief Description**
Bacteriocin production	*nisA*/*nisI* (nisin) *salA*/*salI* (salivaricin)	Antimicrobial peptides [34]
Lactate dehydrogenases	*ldhL* (l-lactate dehydrogenase)	Critical enzyme for lactic acid fermentation, affecting taste and shelf-life of fermented products [35].
Peroxide biosynthesis	*pox* (pyruvate oxidase) *nox* (NADH oxidase)	Generation of hydrogen peroxide, which can serve as a defense mechanism against pathogens [36].
Antibiotic resistance	*mate* (multi-antimicrobial extrusion protein) *gyrB* (DNA gyrase subunit B) *gyrA* (DNA gyrase subunit A)	Genes that may confer intrinsic resistance to some antibiotics [37].
Bile salt hydrolases	*bsh* (bile salt hydrolase)	Key enzyme for bacterial survival in the gastrointestinal tract by deconjugating bile acids [38].
Phage proteins	*int* (phage integrase) *portal* (phage portal protein)	Components of prophages that may impact bacterial genetics and the stability of bacterial populations in fermented foods [39].
Stress response (thermal stress)	*dnaK* (chaperone DnaK) *dnaJ* (chaperone DnaJ) *groEL* (chaperonin GroEL) *groES* (co-chaperonin GroES)	Proteins that assist in the proper folding of other proteins and stress recovery, essential for survival at high temperatures [40].
Stress response (oxidative stress)	*gshA* (glutamate-cysteine ligase) *grx* (glutaredoxin)	Enzymes that participate in the cellular response to oxidative stress by synthesizing antioxidants and repairing damaged proteins [40].
Stress response (osmotic stress)	*glpF* (glycerol uptake facilitator), proP (propanediol diffusion facilitator)	Proteins that help bacteria cope with high osmolarity environments by regulating the uptake of compatible solutes [40].
Proteases	*glpG* (Rhomboid protease) *clpX* (ATP-dependent Clp protease ATP-binding subunit)	Proteases that degrade misfolded proteins, influencing texture and flavor maturation in fermented products [41,42].
Peptidases	*pepT* (Peptidase T) *pepD* (Dipeptidase)	Enzymes that cleave peptides into amino acids, affecting the taste profile of fermented foods by releasing flavor precursors [41,42].
Aromatic pathways	*aroD* (3-dehydroquinate dehydratase) *qsuB* (quinate 5-dehydrogenase)	Enzymes involved in the synthesis of aromatic amino acids, precursors to a variety of flavor and aroma compounds in foods [43].

## Data Availability

Whole genome sequences of *L. salivarius* SP36 were deposited in GenBank under the accession number SAMN40261862. The rest of the original contributions presented in the study are included in the article, further inquiries can be directed to the corresponding author.
